# Inhibition of histone deacetylation with vorinostat does not prevent tunicamycin-mediated acute kidney injury

**DOI:** 10.1371/journal.pone.0260519

**Published:** 2021-11-30

**Authors:** Rachel E. Carlisle, Salwa Farooqi, Ming Chan Zhang, Sarah Liu, Chao Lu, Andy Phan, Elise Brimble, Jeffrey G. Dickhout

**Affiliations:** 1 McMaster University and St. Joseph’s Healthcare Hamilton, Hamilton, Ontario, Canada; 2 Department of Medicine, University of Toronto, Toronto, Ontario, Canada; National Institutes of Health, UNITED STATES

## Abstract

Endoplasmic reticulum (ER) stress is associated with acute kidney injury (AKI) caused by various mechanisms, including antibiotics, non-steroidal anti-inflammatory drugs, cisplatin, and radiocontrast. Tunicamycin (TM) is a nucleoside antibiotic that induces ER stress and is a commonly used model of AKI. 4-phenylbutyrate (4-PBA) is a chemical chaperone and histone deacetylase (HDAC) inhibitor and has been shown to protect the kidney from ER stress, apoptosis, and structural damage in a tunicamycin model of AKI. The renal protection provided by 4-PBA is attributed to its ability to prevent misfolded protein aggregation and inhibit ER stress; however, the HDAC inhibitor effects of 4-PBA have not been examined in the TM-induced model of AKI. As such, the main objective of this study was to determine if histone hyperacetylation provides any protective effects against TM-mediated AKI. The FDA-approved HDAC inhibitor vorinostat was used, as it has no ER stress inhibitory effects and therefore the histone hyperacetylation properties alone could be investigated. *In vitro* work demonstrated that vorinostat inhibited histone deacetylation in cultured proximal tubular cells but did not prevent ER stress or protein aggregation induced by TM. Vorinostat induced a significant increase in cell death, and exacerbated TM-mediated total cell death and apoptotic cell death. Wild type male mice were treated with TM (0.5 mg/kg, intraperitoneal injection), with or without vorinostat (50 mg/kg/day) or 4-PBA (1 g/kg/day). Mice treated with 4-PBA or vorinostat exhibited similar levels of histone hyperacetylation. Expression of the pro-apoptotic protein CHOP was induced with TM, and not inhibited by vorinostat. Further, vorinostat did not prevent any renal damage or decline in renal function caused by tunicamycin. These data suggest that the protective mechanisms found by 4-PBA are primarily due to its molecular chaperone properties, and the HDAC inhibitors used did not provide any protection against renal injury caused by ER stress.

## Introduction

Acute kidney injury (AKI) is a contributor to the Global Burden of Disease (defined by the World Health Organization), and can severely impair the function and damage the structure of the kidney [1]. AKI can occur due to pre-renal, renal or post-renal causes; the most common include ischemia [2, 3], nephrotoxic drugs [4], and radiocontrast medium [5]. These damaging insults cause acute tubular necrosis, which leads to tubular atrophy, loss of the brush border of the renal tubule, and cellular vacuolization of the tubular epithelium. The treatments for AKI are limited to renal replacement therapy in severe cases, and supportive therapy in cases that do not reach the threshold for dialysis. However, an event of AKI increases a patient’s risk of chronic kidney disease, end stage renal disease, and premature death [6]. This illustrates the need for research to elucidate viable prevention and treatment options of AKI for patients.

Tunicamycin (TM) has been used as a model of nephrotoxic intrinsic AKI [7]. TM is a known endoplasmic reticulum (ER) stress inducer. It induces ER stress by preventing N-linked glycosylation, thereby increasing expression of ER stress response proteins. The expression of these ER stress response proteins has been associated with tubular damage and apoptosis in the kidney [8]. We have shown that low dose tunicamycin primarily damages the pars recta of the kidney and induces ER stress and apoptosis in this region. Further, we have demonstrated that inhibiting ER stress with 4-phenylbutyrate (4-PBA) partially prevents renal injury, and C/EBP homologous protein (CHOP) knock out mice treated with TM do not develop renal injury [8].

4-PBA is a low molecular weight protein-folding chaperone, a weak histone deacetylase (HDAC) inhibitor, and an ammonia scavenger [9]. 4-PBA acts as a protein-folding chaperone, which can inhibit ER stress. Misfolded proteins typically have exposed hydrophobic regions, which interact with the hydrophobic regions of 4-PBA [10, 11]; this encourages the proteins to fold in their proper conformation by preventing the formation of irreversible protein aggregates. HDAC inhibitors affect the expression of genes associated with renal injury by modulating chromatin structure. HDACs remove acetyl groups from histones and induce high-affinity binding of DNA to deacetylated histones. This condenses the DNA structure and prevents gene transcription. HDAC inhibitors prevent the deacetylation of histones and alter the expression of genes [12].

The reno-protective effects of 4-PBA have been demonstrated in TM-induced AKI [8], as well as hypertensive models of chronic kidney disease [13, 14]. In these models, 4-PBA was able to reduce expression of the pro-apoptotic protein CHOP, preventing apoptosis and subsequent renal injury. However, the precise mechanism(s) by which 4-PBA provides protection against renal injury have not been determined.

As such, it is not fully understood whether the reno-protective effects of 4-PBA are due entirely to preventing ER stress and ER stress-induced apoptosis or if HDAC inhibition plays a role through modulation of gene expression. ER stress inhibition has shown reno-protective effects, as demonstrated by tauroursodeoxycholic acid (TUDCA) [15], which has no HDAC inhibitory mechanism. However, methyl-4-(phenylthio) butanoate, an HDAC inhibitor, has shown potential in enhancing recovery after AKI [16]. We hypothesized that renal damage induced by TM is caused primarily by ER stress induction; thus, TM-mediated AKI would not be prevented with the HDAC inhibitor vorinostat.

## Materials and methods

### Animal study

Male C57BL/6 wild type mice were used in this experiment. Mice were maintained at McMaster University with free access to food and water. Mice were housed in a 12-hour light-dark cycle and were 14-weeks old at the onset of this experiment. All animal work was done in accordance with and approved by the McMaster University Animal Research Ethics Board.

Mice were randomly allocated to one of four groups: 1) sham; 2) tunicamycin (TM; 0.5 mg/kg); 3) TM with 4-phenylbutyrate (1 g/kg/day) in the drinking water (TM+PBA); or 4) TM with vorinostat (50 mg/kg/day) oral gavage (TM+vor). 4-PBA and vorinostat were provided for ten days. Mice were intraperitoneally injected with TM on day seven and sacrificed on day ten. At sacrifice, blood and kidneys were collected for analysis.

### Cell culture

Immortalized human proximal tubule epithelial (HK-2) cells were used for most *in vitro* work. Cells were initially purchased from ATCC. HK-2 cells stably transfected with XBP1sVenus reporter were also used [17, 18]. These cells express a FLAG-tag when XBP1 is spliced. All cells were grown in a culture medium of 1:1 low glucose Dulbecco’s modified eagle medium and F12 GlutaMAX nutrient mix (with 1% penicillin/streptomycin and 10% fetal bovine serum) unless otherwise specified.

### Reagents

The following reagents and doses were used for *in vitro* experiments. Tunicamycin was used at a dose of 1 μg/ml. 4-PBA was used at a dose of 1 mM. Vorinostat was used at a dose of 5 μM. TUDCA was used at a dose of 500 μM. 4μ8c was used at a dose of 10 μM. Trichostatin A was used at doses varying from 5 nM to 1200 nM.

### Protein aggregation

Thioflavin T was used as an indicator of protein aggregation in cells at a dose of 5 μM. HK-2 cells were grown on coverslips, and treated in triplicate with DMSO (veh), TM, TM with 4-PBA (TM+PBA), or TM with vorinostat (TM+vor) for 24 hrs. Cells were subsequently incubated with Thioflavin T for 15 mins at 37°C in fresh media. Cells were then washed with PBS, fixed with 4% paraformaldehyde overnight at 4°C, and mounted on microscope slides. Slides were imaged using an Olympus IX81 Nipkow scanning disc confocal microscope and quantified using MetaMorph image analysis software. Quantification was performed to evaluate relative fluorescence of aggregated proteins, as previously [19].

### Periodic acid-Schiff staining and injury score

Periodic acid-Schiff (PAS) staining was performed, as previously described in our study [8]. Briefly, slides were de-paraffinized in xylene and ethanol baths, oxidized in 1% aqueous periodic acid, treated with Schiff reagent, and counter-stained in haematoxylin. Slides were dehydrated in a graded series of ethanol and placed in xylene baths for coverslip mounting using Permount. PAS-stained kidneys were scored for injury as follows: 0, 0% kidney damage; 1, 1–25% kidney damage; 2, 26–50% kidney damage; 3, 51–75% kidney damage; 4, >75% kidney damage. Renal damage was characterized by loss of tubular brush border and epithelial cell nuclei, and/or vacuolization of the tubular epithelial cells. Previous work has demonstrated that TM-induced injury occurs primarily in the pars recta of the kidney at the dose used, and thus that was the region scored for injury.

### Immunofluorescent and immunohistochemical tissue staining

Immunofluorescent staining was performed, as previously described in our study [8]. Briefly, slides were de-paraffinized as described above, and blocked in normal donkey serum. Primary antibody against acetyl H3 (Millipore #06–599; 1:200) was incubated for 1 hr, and secondary antibody (goat anti-rabbit, 594 nm; 1:200) was incubated for 30 mins. Slides were then incubated in DAPI to visualize the nuclei and mounted in PermaFluor.

Immunohistochemical staining was performed, as previously [14]. After de-paraffinization, slides were incubated with endogenous peroxidase, and antigen retrieval was performed (citric acid buffer for 1 hr). Slides were then blocked in normal goat serum, incubated in the primary antibody for 1 hr (CHOP; sc-575; 1:40), and incubated in a biotinylated secondary antibody (goat anti-rabbit; 1:500) for 30 mins. Streptavidin/peroxidase and NovaRed were used to visualize the protein of interest. Slides were mounted in Permount.

Quantification of acetyl histone 3 (H3)- and CHOP-stained slides was performed using ImageJ software. Images were separated by colour, retaining the ‘red’ image (H3 or CHOP) and blue image (DAPI), which displayed the staining most clearly. Thresholding the image was able to determine which areas were most brightly stained, and the software was then able to analyze the particles to determine how many nuclei were stained. The number of H3-stained particles was divided by the number of DAPI-stained particles (total cell count), which provided a % of total cells that were stained with H3. CHOP-stained cells were not divided by total cell count but are presented as ‘number of CHOP-positive cells per high-powered field’.

### Plasma analysis

Serum creatinine was measured using an enzymatic assay (Pointe Scientific), as per the manufacturer’s instructions. Briefly, serum was incubated in a 96-well plate with ‘reagent 1’ for 5 mins at 37°C, after which the absorbance was read at 550 nm. ‘Reagent 2’ was then added to each well, and the plate was again incubated for 5 mins at 37°C. Absorbance was read a second time, and the change in absorbance values (A2-A1) was used to quantify serum creatinine levels. Creatinine standard (221 μmol/L) was purchased from Pointe Scientific (C7513-STD), and additional standards were produced via dilution (150, 100, 50, 25, 10 μmol/L) to make a standard curve. Changes in absorbance values from unknown samples were interpolated into the standard curve.

### Cell death assays

#### Lactate dehydrogenase assay

LDH assay was performed as per the manufacturer’s instructions (BioLegend). Briefly, cells were synchronized in media containing 1% FBS for 24 hrs, at which point the cells were washed and new media (1% FBS) was added and cells were treated. Media was collected at 24 hrs and 48 hrs for analysis. Cells were treated with 1% Triton-X, a non-ionic detergent that results in cell lysis, to produce a positive control. Media from treated cells and the ‘working reagent solution’ were added to a 96-well plate. After 30 mins of incubation, the ‘stop solution’ was added. Absorbance was read at 490 nm using a colorimetric spectrophotometer. Cell death (%) was calculated using the 1% Triton-X high control (100% cell death) and low control (untreated media; 0% cell death) in the following equation:

$$\text{Cytotoxicity}\;\left(\%\right)=\left(\left(\text{test}\;\text{substance}–\text{low}\;\text{control}\right)/\left(\text{high}\;\text{control}–\text{low}\;\text{control}\right)\right)*100$$

#### TUNEL staining

A terminal deoxynucleotidyl transferase dUTP nick end labelling (TUNEL) staining kit was used to stain cells undergoing apoptosis, as per the manufacturer’s instructions (TMR-in situ cell death detection kit; Roche) [8]. Apoptotic (green) cells and total (blue) cells were counted and analyzed using ImageJ software. Cells were considered apoptotic if the emission of the TMR-oligo tag overlapped with the DAPI nuclear stain. Apoptosis was expressed as a percentage of total cells. 10 images of 3 slides were examined for each treatment.

### Gel electrophoresis

Western blots were performed with cell lysates, as well as renal tissue lysates, all in triplicate. 4X SDS lysis buffer with protease and phosphatase inhibitors was used for cell or tissue lysis. A protein assay was performed prior to Western blotting (DC Protein Assay; BioRad). A 15% SDS-PAGE gel was used to probe for acetyl H3, and 10% SDS-PAGE gels were used for all other proteins. Primary antibodies were detected using a horseradish peroxidase-bound secondary antibody and enhanced chemiluminescence. Acetyl H3 (#06–599; Millipore) was used at a dilution of 1:1000, CHOP (sc-7351; Santa Cruz) was used at a dilution of 1:200, β-actin (#66009–1; ProteinTech) was used at a dilution of 1:5000, and GAPDH (#2118; Cell Signaling) was used at a dilution of 1:1000. GRP78 was measured using a KDEL antibody (SPA-827; Stressgen), which binds to the KDEL amino acid sequence in GRP78. It was used at a dilution of 1:1000. Anti-mouse and anti-rabbit secondary antibodies were used at 1:5000 (BioRad). Results were densitometrically quantified using ImageLab software, and each protein was expressed as a ratio of β-actin or GAPDH loading control.

### Statistical analysis

Statistical analysis was performed using GraphPad Prism software. Results were analysed using a student’s T-test or one-way analysis of variance with Tukey’s post-hoc test, as appropriate. Results are expressed as the mean +/- standard error of the mean. Significance was recognized at the 95% level.

## Results

### Vorinostat does not prevent endoplasmic reticulum stress in vitro

**Fig 1:**
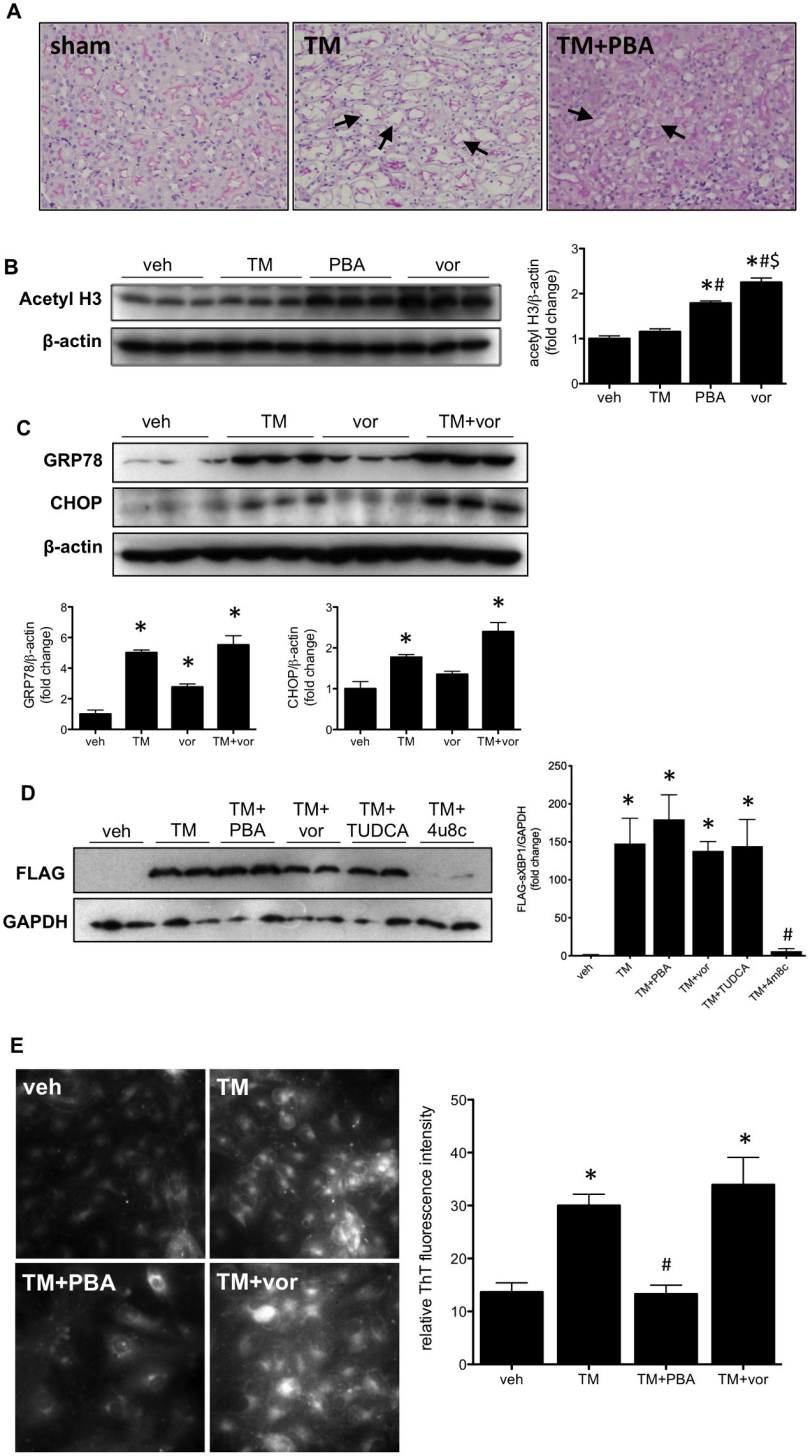
(A) Wild type mice were given tunicamycin (TM; 0.5 mg/kg I.P) for 3 days with or without 4-phenylbutyrate (PBA; 1 g/kg/day). Kidneys were stained with periodic acid-Schiff reagent to examine structural injury. Injury in the pars recta is indicated with arrows. (B) Human proximal tubular (HK-2) cells were treated with tunicamycin (TM), PBA, or vorinostat (vor) for 4 hrs. Acetylated histone 3 (H3) was increased in cells treated with PBA or vorinostat. (C) HK-2 cells were treated with TM, vor, or TM+vor for 24 hrs. Western blotting demonstrates increased GRP78 and CHOP in response to TM treatment. Vorinostat alone only increased GRP78 expression. (D) FLAG-HK-2 cells were treated with TM in the absence or presence of PBA, vorinostat, TUDCA, or 4μ8C for 18 hrs. TM increased FLAG expression (indicating XBP1 splicing), and 4μ8C was able to inhibit this effect. (E) HK-2 cells were treated with TM with PBA or vorinostat for 24 hrs and stained with thioflavin T. Protein aggregation was increased with TM treatment, and reduced with PBA. *, p<0.05 vs veh; #, p<0.05 vs TM; $, p<0.05 vs PBA.

Wild type mice treated with TM develop acute kidney injury in the pars recta of the kidney. Damage in the pars recta is characterized by atrophy of the tubules, loss of proximal tubule brush border, and vacuolization of proximal tubular epithelial cells. As reported previously [8], co-treatment with the low molecular weight chemical chaperone 4-PBA prevents this damage (Fig 1A). To determine if the protective effects of 4-PBA treatment were due to its HDAC inhibitory effects, HK-2 cells were treated with TM, 4-PBA, or vorinostat. TM treatment did not alter H3 acetylation; however, both 4-PBA and vorinostat increased H3 acetylation (Fig 1B), indicating their HDAC inhibitory effects. HK-2 cells treated with TM and vorinostat were examined for markers of ER stress. Vorinostat did not affect TM-mediated GRP78 or CHOP expression, though vorinostat alone increased GRP78 expression (Fig 1C). Further, TM-induced spliced XBP1 (FLAG) was not inhibited by 4-PBA, vorinostat, or TUDCA. Splicing of XBP1 was only prevented by the IRE1 inhibitor 4μ8c (Fig 1D). Thioflavin T staining demonstrated increased protein aggregation with TM treatment, which was prevented with the protein-folding chaperone 4-PBA; however, vorinostat had no effect on protein aggregation (Fig 1E).

### Vorinostat increases cell death in vitro

**Fig 2:**
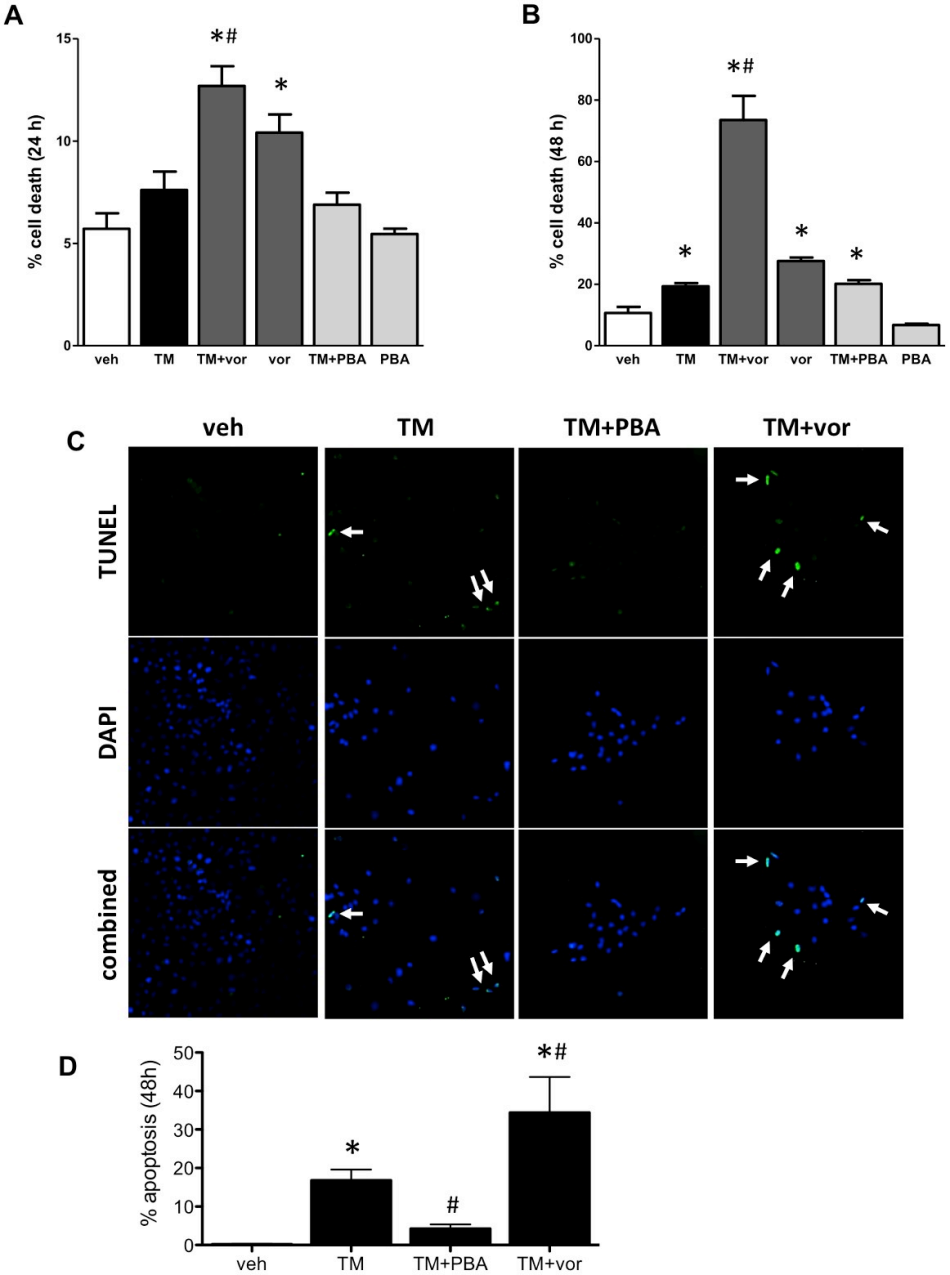
(A) Human proximal tubular (HK-2) cells were treated with tunicamycin (TM), TM with 4-phenylbutyrate (TM+PBA), TM with vorinostat (TM+vor), 4-phenylbutyrate alone (PBA), or vorinostat alone (vor) for 24 hrs. TM+vor and vor increased cell death. (B) After 48 hrs, TM increased cell death, which was not inhibited by PBA. Vorinostat increased cell death, and exacerbated TM-mediated cell death. (C) Cells were also stained for apoptotic cell death (green; arrows) after 48 hrs. TM induced apoptosis, which was inhibited by PBA. Vorinostat exacerbated TM-induced apoptotic cell death. *, p<0.05 vs veh; #, p<0.05 vs TM.

HK-2 cells were treated with TM in the presence or absence of 4-PBA or vorinostat. Cells were also treated with 4-PBA alone and vorinostat alone. Total cell death was measured at 24 and 48 hrs. At 24 hrs, cell death was increased in cells treated with TM+vor or vor alone. TM alone or in combination with 4-PBA did not affect cell death at 24 hrs (Fig 2A). At 48 hrs, TM induced cell death, which was exacerbated by vorinostat co-treatment. Vorinostat alone also induced cell death. 4-PBA alone did not affect cell death and did not prevent TM-mediated cell death (Fig 2B). Apoptotic cell death was increased in TM-treated cells at 48 hrs. Apoptosis was prevented with 4-PBA co-treatment, but worsened with vorinostat co-treatment (Fig 2C).

### Vorinostat does not inhibit CHOP expression in the kidney

**Fig 3:**
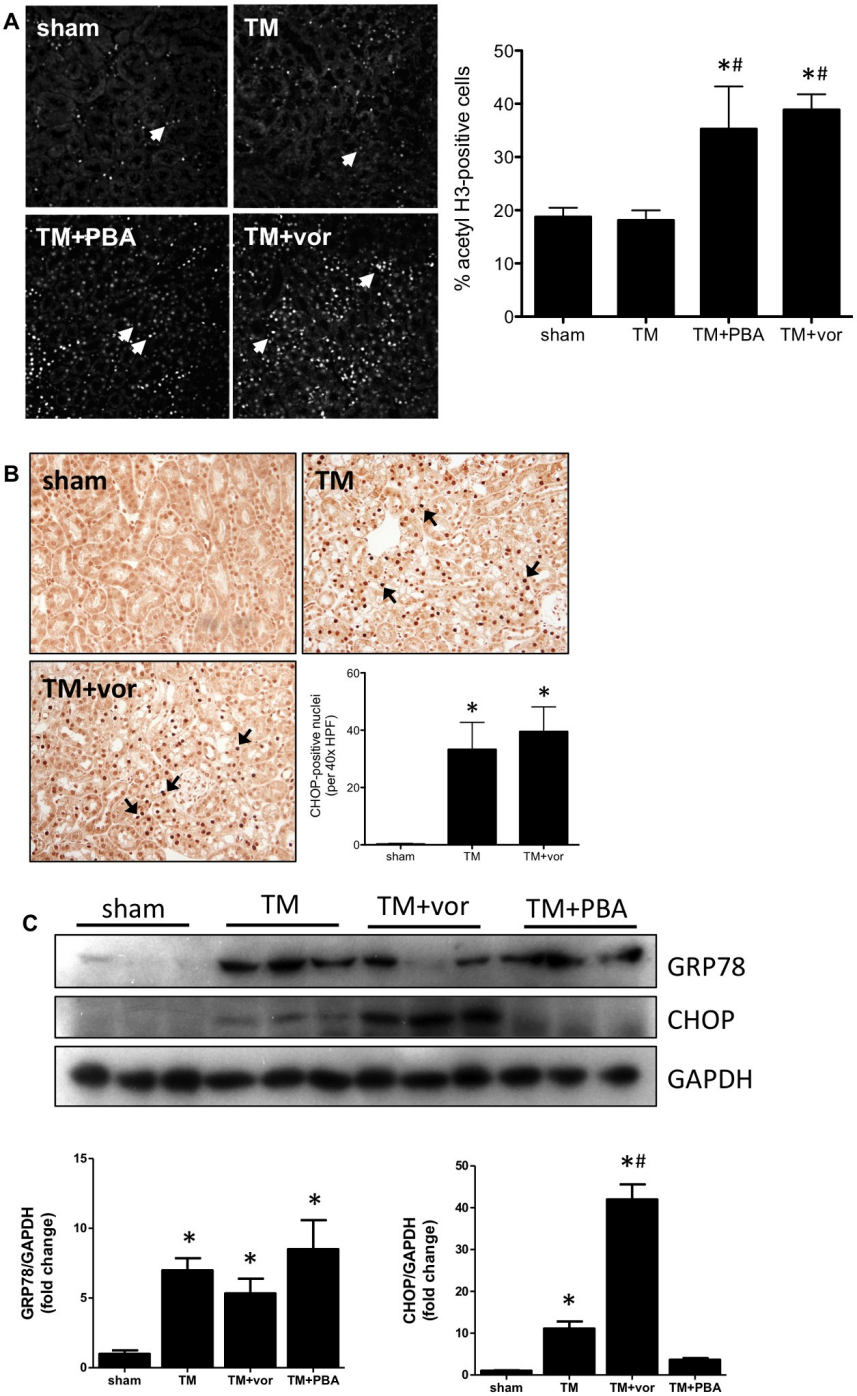
Wild type mice were given tunicamycin (TM; 0.5 mg/kg I.P) for 3 days with or without PBA (1 g/kg/day) or vorinostat (vor; 50 mg/kg/day). (A) Kidneys were stained for acetylated histone 3 (H3; arrows). H3 acetylation was increased in TM+PBA and TM+vor mice. (B) Kidneys were also stained for CHOP (arrows). TM induced CHOP expression, which was not prevented by vorinostat. (C) The pars recta of the kidney was isolated and underwent Western blotting for GRP78 and CHOP. GRP78 is increased in TM-treated kidneys, and not inhibited by vorinostat or 4-PBA. CHOP expression is increased in both TM and TM+vor kidneys. *, p<0.05 vs sham; #, p<0.05 vs TM.

Wild type mice were treated with TM bolus injection (0.5 mg/kg) and examined for renal pathology three days later, with or without 4-PBA or vorinostat co-treatment. Kidneys were stained for acetyl H3; similar to our *in vitro* data, TM did not affect H3 levels in the kidney. Both 4-PBA and vorinostat increased H3 acetylation (Fig 3A) demonstrating their HDAC inhibitor effects. Further, CHOP expression was significantly increased in TM-treated mice compared with sham-treated mice. The staining was primarily evident in the region of damage, the pars recta of the kidney. Interestingly, mice co-treated with vorinostat did not exhibit any difference in CHOP expression, when compared with TM-treated mice (Fig 3B). Western blotting was performed to examine ER stress markers in the kidney. TM induced both GRP78 and CHOP expression. Increased GRP78 expression was not prevented by vorinostat or PBA. Vorinostat increased CHOP expression further than TM alone, while PBA prevented CHOP expression (Fig 3C).

### Vorinostat did not prevent TM-mediated acute kidney injury

**Fig 4:**
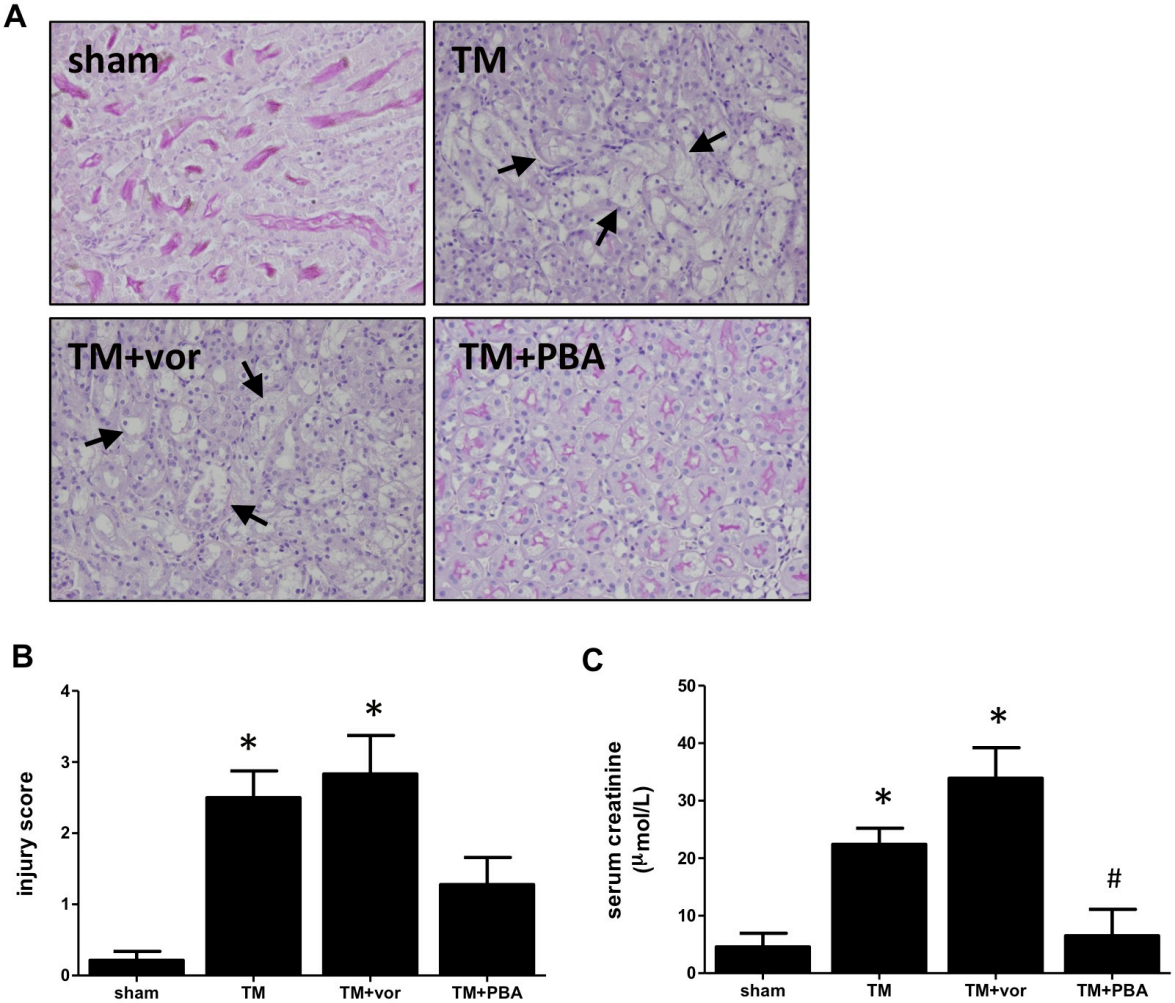
Wild type mice were given tunicamycin (TM; 0.5 mg/kg I.P) for three days with or without vorinostat (vor; 50 mg/kg/day) or 4-phenylbutyrate (PBA; 1 g/kg/day). (A) Kidneys were PAS stained to examine structural damage in the pars recta. Damage is indicated with arrows. (B) Kidney injury scores demonstrate TM caused significant damage in the pars recta, with TM+vor-treated mice having similar levels of damage. (C) Serum creatinine levels were increased in TM-treated mice. Vorinostat did not affect TM-induced creatinine levels, which was prevented by PBA. *, p<0.05 vs sham; #, p<0.05 vs TM.

TM-treated mice exhibit kidney damage in the pars recta of the kidney, including tubular atrophy, loss of brush border, and epithelial cell vacuolization. Co-treatment with vorinostat did not prevent renal damage from occurring in these mice (Fig 4A). Quantification was performed as described in the methods (Fig 4B). Further, mice treated with TM developed impaired renal function, as evidenced by increased serum creatinine. Co-treatment with vorinostat did not lower serum creatinine levels that were elevated by TM treatment, while PBA co-treatment did (Fig 4C).

### Trichostatin A does not prevent endoplasmic reticulum stress in proximal tubular cells

**Fig 5:**
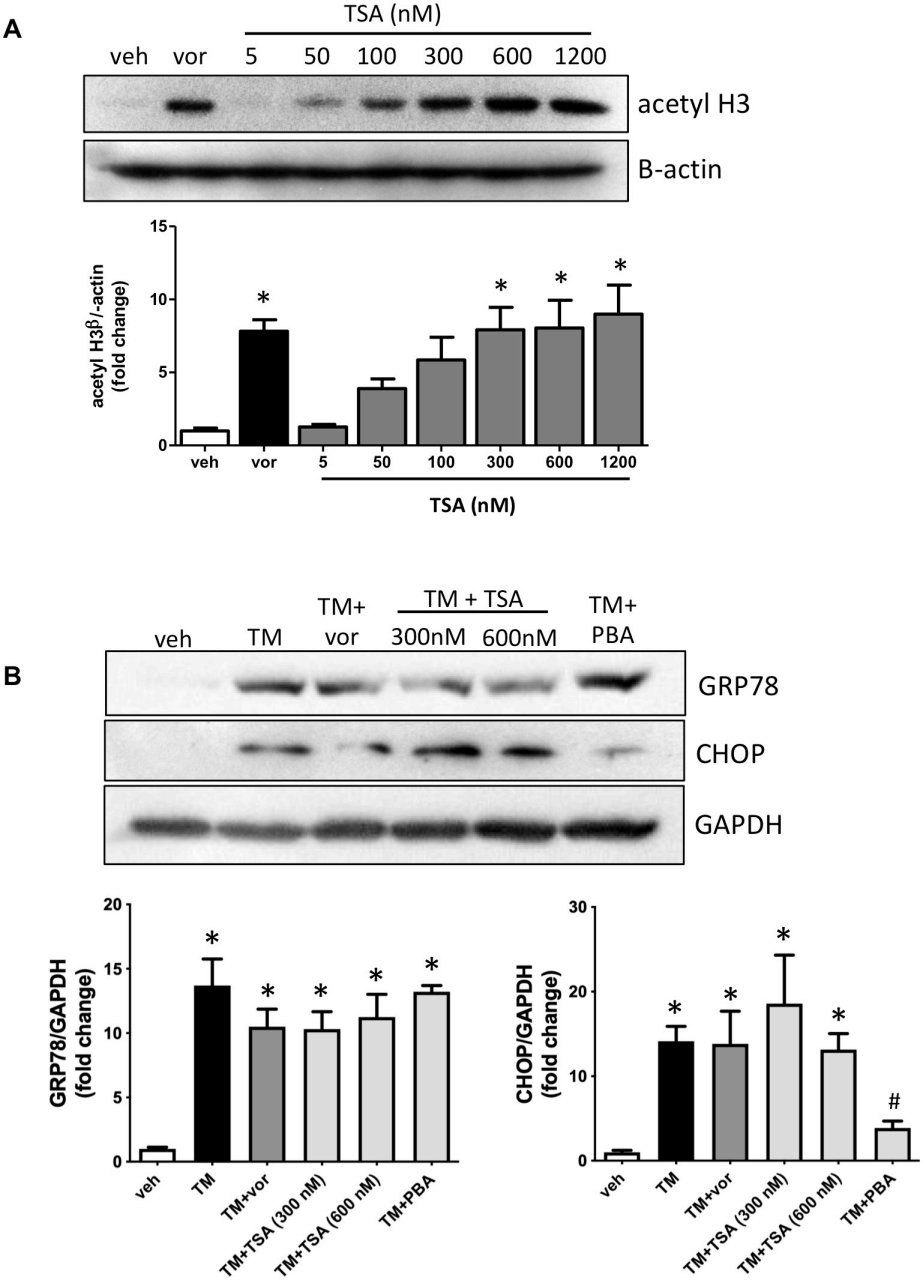
(A) Human proximal tubular (HK-2) cells were treated with vorinostat (vor) or varying doses of trichostatin A (TSA) for 4 hrs. Western blotting demonstrated that 300 nM, 600 nM and 1200 nM TSA produced similar levels of H3 acetylation as vorinostat. (B) HK-2 cells were treated with tunicamycin (TM) with or without vor, TSA or PBA for 24 hrs. Western blotting demonstrates that vor, TSA and PBA do not reduce TM-mediated GRP78 induction, and only PBA prevents TM-induced CHOP expression. *, p<0.05 vs veh; #, p<0.05 vs TM.

To determine similar levels of HDAC inhibition with an additional HDAC inhibitor, HK-2 cells were treated with our treatment dose of vorinostat and varying doses of trichostatin A (TSA). Cells then underwent western blotting for acetyl H3, and quantification determined that the doses of 300 nM, 600 nM and 1200 nM TSA produced results similar to 5 μM vorinostat. The doses of 300 nM and 600 nM were most similar and were subsequently chosen to be used for further experiments (Fig 5A). To examine the ER stress inhibitory effects of HDAC inhibitors, HK-2 cells were treated with TM in the presence or absence of vorinostat, 300 nM TSA, 600 nM or PBA. Western blotting demonstrates that none of the HDAC inhibitors prevent GRP78 expression *in vitro*. Neither vorinostat nor TSA prevent CHOP expression, while PBA does (Fig 5B).

### Trichostatin A does not prevent tunicamycin-induced cell death

**Fig 6:**
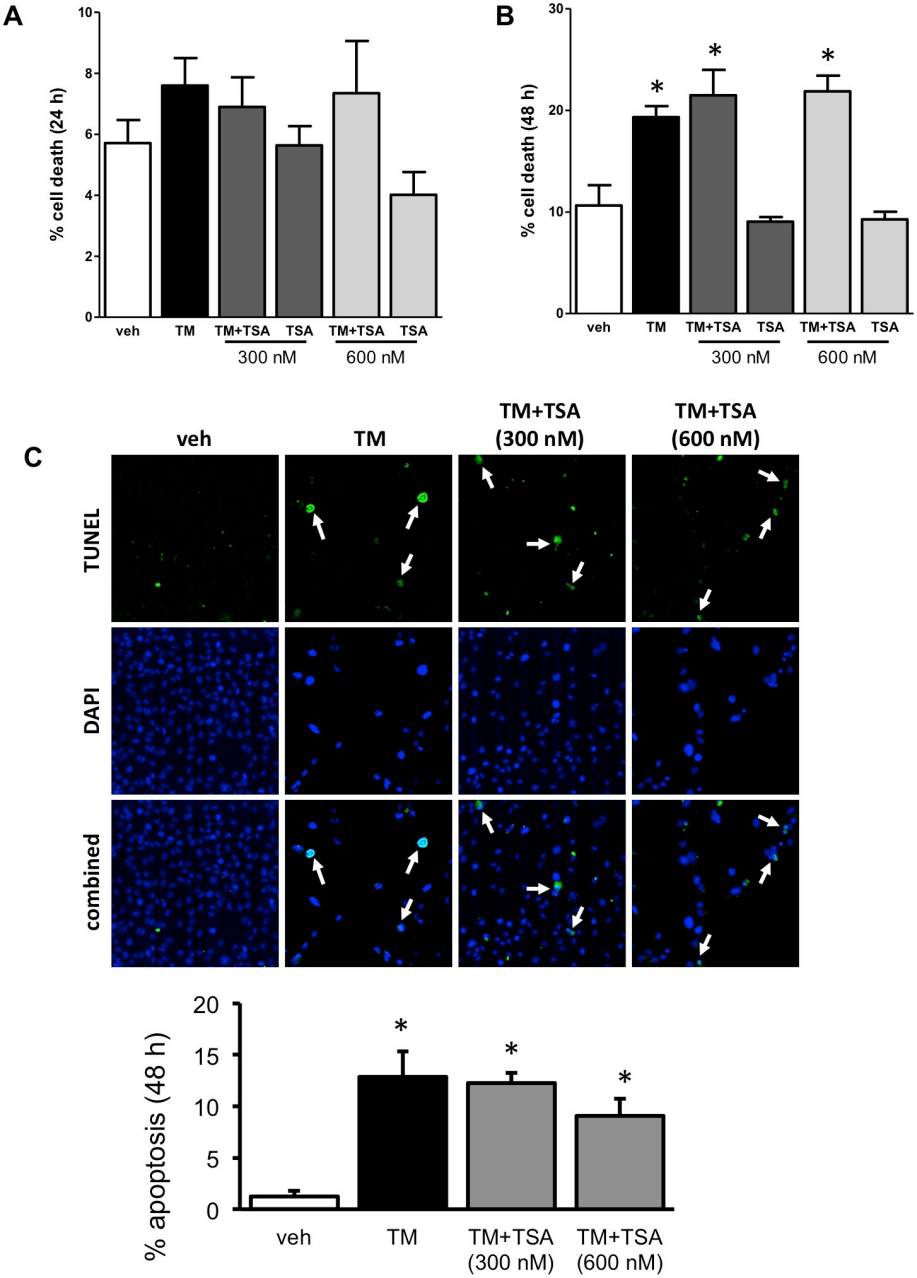
Human proximal tubular cells were treated with TM with TSA (300 nM or 600 nM). (A) No significant cell death was found at 24 hrs. (B) At 48 hrs, TM induced cell death, which was not prevented by co-treatment of TSA. (C) At 48 hrs, TM induced apoptosis, which was not prevented by co-treatment of TSA. *, P<0.05 vs veh.

To determine the effect of TSA on proximal tubular cell death, HK-2 cells were treated with TM and the HDAC inhibitor. At 24 hrs, no treatment had any effect on cell death (Fig 6A). At 48 hrs, TM significantly induced cell death, which was not prevented with TSA co-treatment (Fig 6B). Additionally, TUNEL staining determined that TSA does not prevent TM-mediated apoptosis (Fig 6C).

## Discussion

TM is a commonly used *in vitro* model of ER stress and an *in vivo* model of intrinsic AKI. We, and others, have previously shown that TM causes damage to the pars recta of the kidney [8]. With TM treatment, renal tubules are atrophied, there is a loss of brush border, and proximal tubular cells become vacuolized. Our previous work demonstrates that the region of damage exhibits markers of ER stress, including GRP78 and CHOP, as well as apoptosis. As 4-PBA is a protein-folding chaperone, our previous work examined the effects of protein aggregation inhibition on TM-induced AKI. Treating mice with the molecular chaperone and HDAC inhibitor 4-PBA partially prevented renal damage, as well as reduced ER stress and apoptosis in the kidney [8].

In addition to 4-PBA, there are a number of other molecular chaperones that can be used to prevent protein aggregation and ER stress. One commonly studied chaperone is TUDCA, a taurine-conjugated derivative of the endogenous bile acid ursodeoxycholic acid [20]. TUDCA has been shown to reduce ER stress and improve glucose homeostasis in a model of diabetes [20], as well as protect against cholestatic liver diseases [21]. Additionally, 4-(4-methoxyphenyl)butyric acid (4-MPBA), a derivative of 4-PBA, shares the protein folding chaperone qualities of 4-PBA, but not the HDAC inhibitor effects [11]. As such, these molecules may provide renal protection against ER stress- and protein misfolding-mediated damage.

In addition to its role as a protein-folding chaperone, 4-PBA is also an HDAC inhibitor; HDAC inhibitors cause histone hyperacetylation by preventing the removal of acetyl groups from histones. The effects of histone acetylation have not been thoroughly examined in TM-induced AKI. Thus, we determined that the pan-HDAC inhibitor vorinostat would be used to examine the effects of HDAC inhibition in this model. Vorinostat was chosen since, like 4-PBA, it is an FDA-approved drug (used to treat cutaneous T cell lymphoma) [22].

Others have demonstrated that HDAC inhibitors can have effects on ER stress, similar to protein-folding chaperones. HDAC inhibitors have been shown to prevent ER stress in bleomycin-treated lung epithelial cells [23], as well as in kidneys of mice with rhabdomyolysis [24] or treated with cisplatin [25]. Interestingly, HDAC inhibitors acetylate spliced XBP1 [26], a transcriptional regulator induced by ER stress. Spliced XBP1 upregulates protein folding chaperones, including GRP78; this would allow a greater protein-folding capacity in the cell, thereby reducing ER stress. In support, our previous *in vitro* work demonstrates that 4-PBA partially inhibits CHOP expression induced by TM, while GRP78 levels remain elevated [8]. The protein folding chaperone GRP78 functions to attenuate the accumulation of unfolded or misfolded proteins; the dampening of ER stress pathway activation is demonstrated by low expression levels of downstream ER stress proteins [8]. Similarly, western blotting from the current study demonstrated increased GRP78 in response to vorinostat treatment. However, vorinostat did not cause any change in expression to other ER stress markers (CHOP, sXBP1) or in protein aggregation *in vitro*. Similarly, TSA did not affect protein levels of GRP78 or CHOP induced by TM in proximal tubular cells. Further, vorinostat did not inhibit expression of GRP78 or CHOP *in vivo*, indicative of absent or inadequate ER stress inhibition.

Studies have demonstrated both anti-apoptotic and pro-apoptotic effects of HDAC inhibition. HDAC inhibitors have been shown to be protective in animal models of kidney disease. In rhabdomyolysis-induced AKI, inhibiting HDAC6 prevented apoptosis, and reduced expression of BAX, BAK, and cleaved caspase-3 [24]. Cisplatin-mediated AKI was prevented with TSA treatment, which reduced cleaved caspase 3 and apoptosis [27]. Cisplatin-mediated apoptosis was also prevented with the HDAC6 inhibitor, 23BB [25]. In contrast, a number of studies demonstrate the pro-apoptotic effects of HDAC inhibition in tumour and cancer cells [28–30]. In cancer cells, vorinostat exhibits cytotoxic effects through caspase-independent mechanisms [31]. Cell death is induced through the mitochondrial death pathway, mediated by increased reactive oxygen species [31]. Further, activation of the MEK/ERK pathway, as well as the inhibition of the JNK pathway can prevent vorinostat-mediated cell death [32]. Additionally, vorinostat induces significant apoptosis in non-cancer cells; cultured rat proximal tubular cells treated with vorinostat undergo cell death caused by both caspase-dependent and caspase-independent mechanisms [33]. These data corroborate our results that *in vitro* treatment with vorinostat induces cell death. Interestingly, tunicamycin also induces formation of reactive oxygen species, leading to cell death [34, 35]. In fact, inhibiting reactive oxygen species prevented tunicamycin-mediated renal injury in old mice [36]. This may provide some insight into the compounding cytotoxic effect of tunicamycin and vorinostat. TSA did not demonstrate any effect on cell death or apoptosis in human proximal tubular epithelial cells, suggesting that the cytotoxic and apoptotic effects of vorinostat are specific to the drug and not due to pan-HDAC inhibition.

HDAC inhibitors possibly exhibit reno-protective effects through a mechanism involving increased autophagy. The commonly used HDAC inhibitors, vorinostat and trichostatin A, induce autophagy in proximal tubular cells [27]. Further, trichostatin A protects renal function and prevents tubular damage caused by cisplatin treatment; the lysosomal inhibitor chloroquine inhibits these protective effects [27]. Of note, tunicamycin itself can induce autophagy in renal cells, and pre-treatment with tunicamycin has reno-protective effects in ischemia-reperfusion-mediated injury. Again, these protective effects are prevented when animals are co-treated with chloroquine [37]. It is possible that, while vorinostat does not prevent ER stress, it can exert reno-protective effects through autophagic mechanisms induced by HDAC inhibition. However, these reno-protective effects were not seen at the time point of our TM model of AKI.

Renal interstitial fibrosis can develop as a downstream result of AKI. Multiple signalling pathways activate the production of fibrotic proteins and cause extracellular matrix components to accumulate in the renal interstitium. While the mechanisms are not fully understood, HDAC inhibition has been shown to prevent the development of renal fibrosis in multiple animal models of disease, including kidneys [38], liver [39], lungs [40], and skin [41]. A class I HDAC inhibitor attenuated unilateral ureteral obstruction-mediated TGF-β1 production, as well as phosphorylation of Smad3 and EGFR [42]. Further, a different class I HDAC inhibitor prevented adriamycin-induced glomerulosclerosis and tubulointerstitial fibrosis [43]. Class III HDAC inhibitors have also blunted the progression of renal fibrosis and inhibited pro-fibrotic signalling pathways [44]. In ischemia-reperfusion-induced AKI, HDAC inhibitor-treated mice were not protected from initial injury (3 days), but were quicker to recover and protected from subsequent development of fibrosis (28 days) [16]. Our previous work demonstrated that there were no fibrotic effects in our TM model of AKI at the 72-hr time point [8], and thus, we did not measure any fibrotic markers in this study. Of note, it has been suggested that HDAC inhibition prevents renal interstitial fibrosis by blunting the inflammatory response in the damaged kidney [45, 46]; in support, tunicamycin is known to induce an inflammatory response in the kidney [47]. Our current study did not examine any effects vorinostat may have on inflammation. While the specific mechanisms behind the anti-fibrotic effects of HDAC inhibition are not fully understood, it is clear that specific and pan-HDAC inhibitors may protect against the development and progression of renal interstitial fibrosis.

While HDAC inhibition has shown protective effects against the decline of renal function and inhibiting renal interstitial fibrosis [24, 27], it is not a significant contributor in preventing renal injury in TM-induced AKI. We previously demonstrated that the HDAC inhibitor and protein-folding chaperone 4-PBA partially prevented TM-induced renal injury, while reducing apoptosis and expression of ER stress markers. While a significant amount of injury was attributed to the induction of ER stress and specifically CHOP, an ER stress protein, the effects of HDAC inhibition were not examined [8]. In this current study, vorinostat did not reduce expression of CHOP *in vitro* or in the kidney, and it was unable to prevent *in vitro* protein aggregation induced by TM. Both PBA and vorinostat increased acetylation within the kidney to similar levels; this suggests that the reno-protective mechanism by 4-PBA was not due to HDAC inhibition, but rather by preventing the accumulation of misfolded proteins. Thus, we conclude that vorinostat-mediated HDAC inhibition does not protect the kidneys from injury due to ER stress.

## Supporting information

S1 Raw images (PDF)
