# Supplementary material for: Inhibition of histone deacetylation with vorinostat does not prevent tunicamycin-mediated acute kidney injury
Source: PLoS One. 2021 Nov 30;16(11):e0260519. doi: 10.1371/journal.pone.0260519 (PMC8631648; doi:10.1371/journal.pone.0260519)

Western blots from Figure 1B

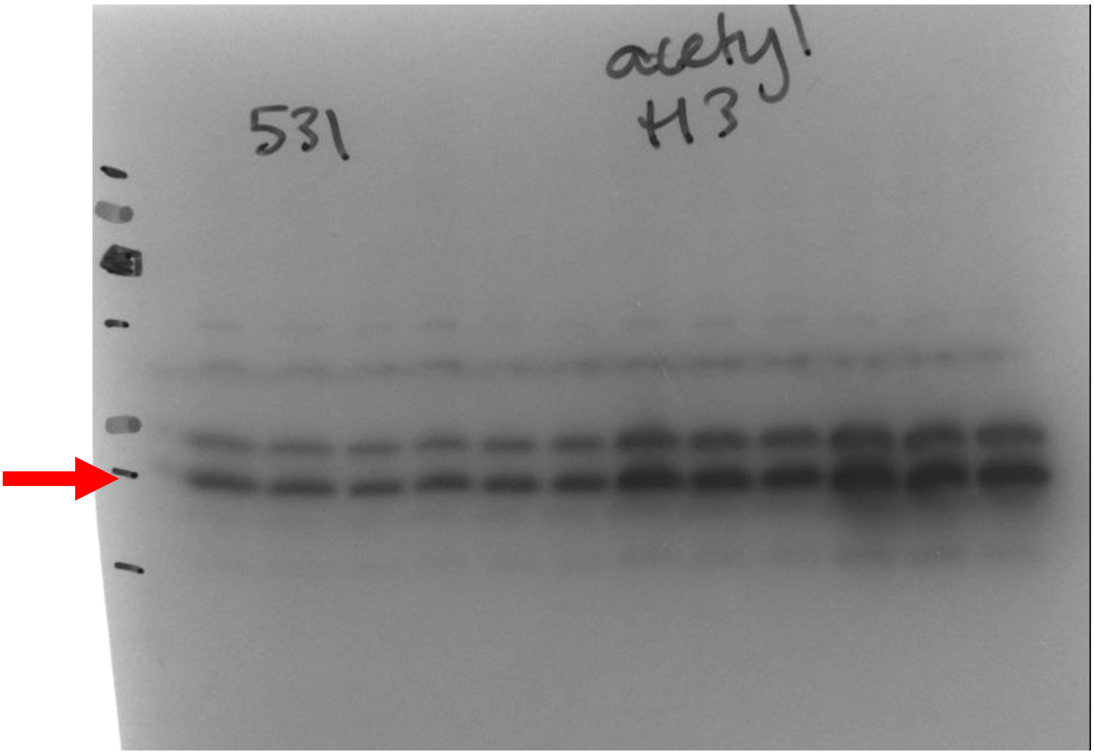

Acetyl H3

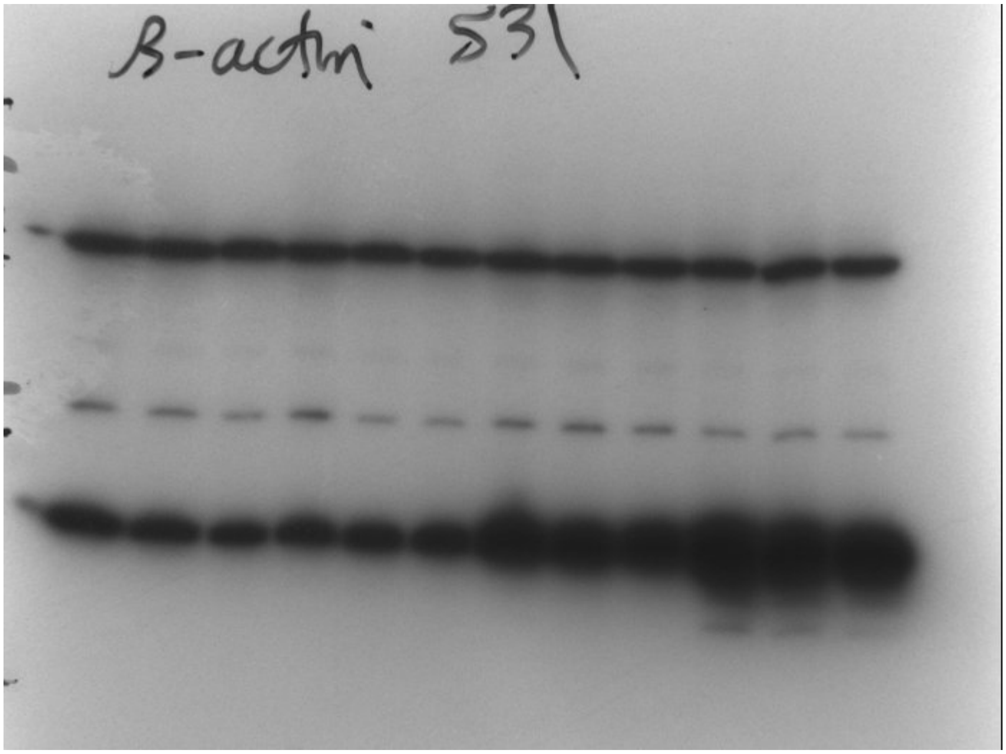

β-actin

Western blots from Figure 1C

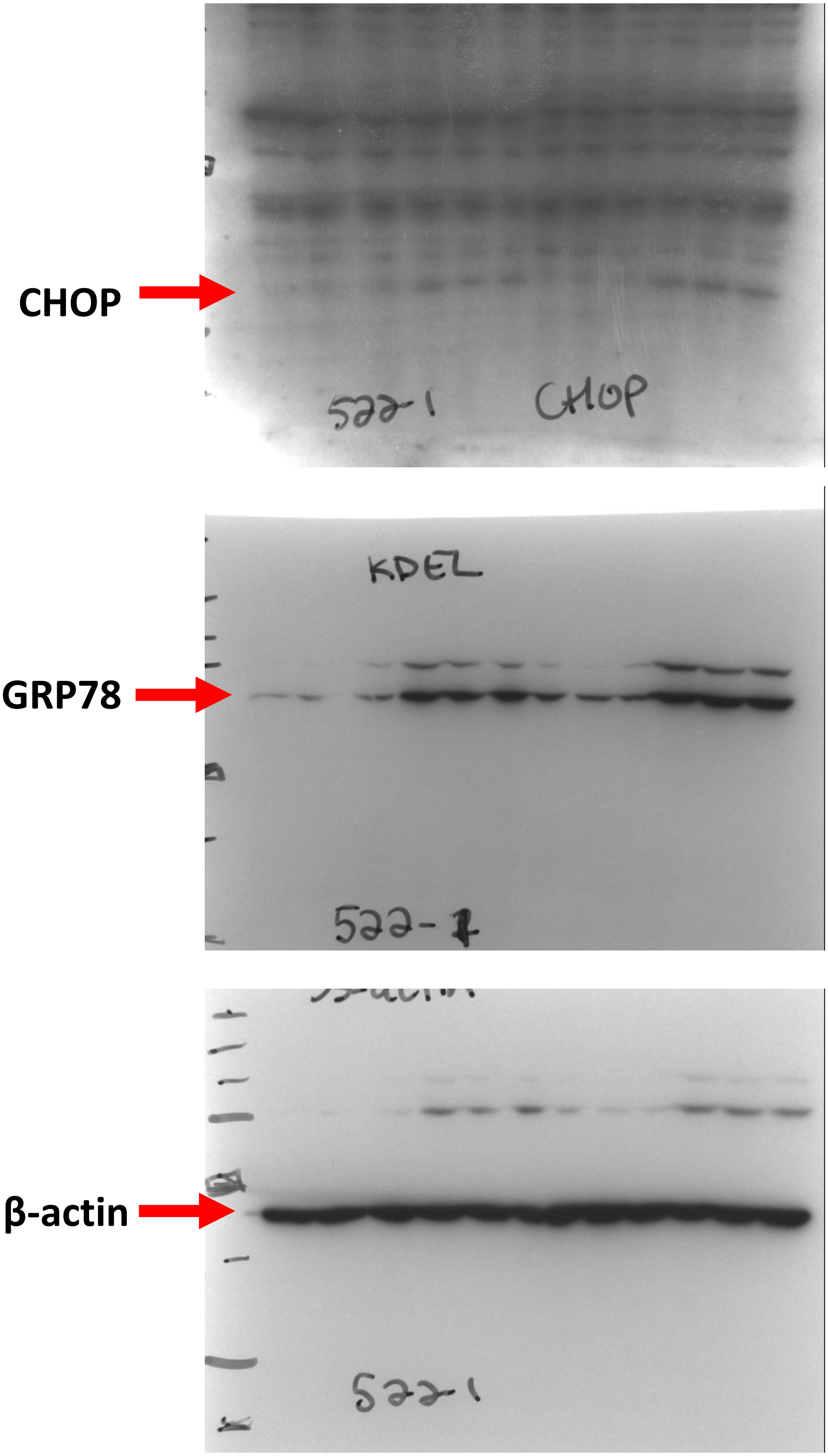

Western blots from Figure 1D

FLAG

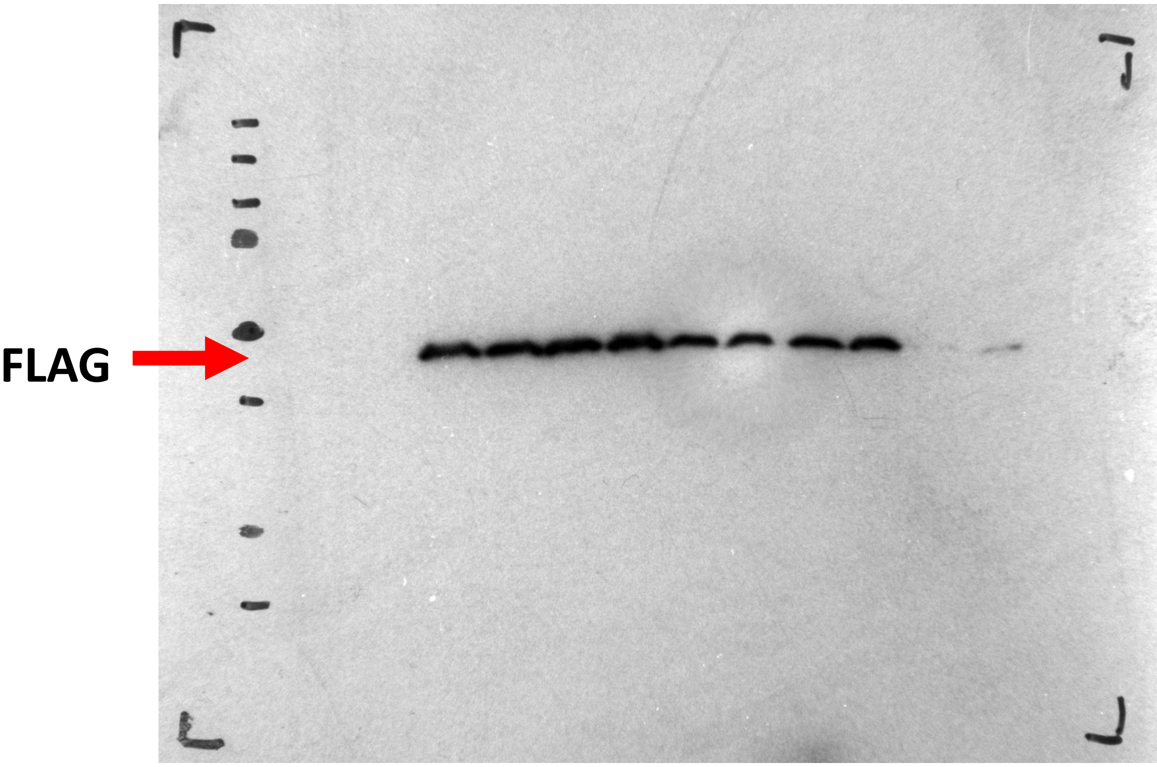

GAPDH

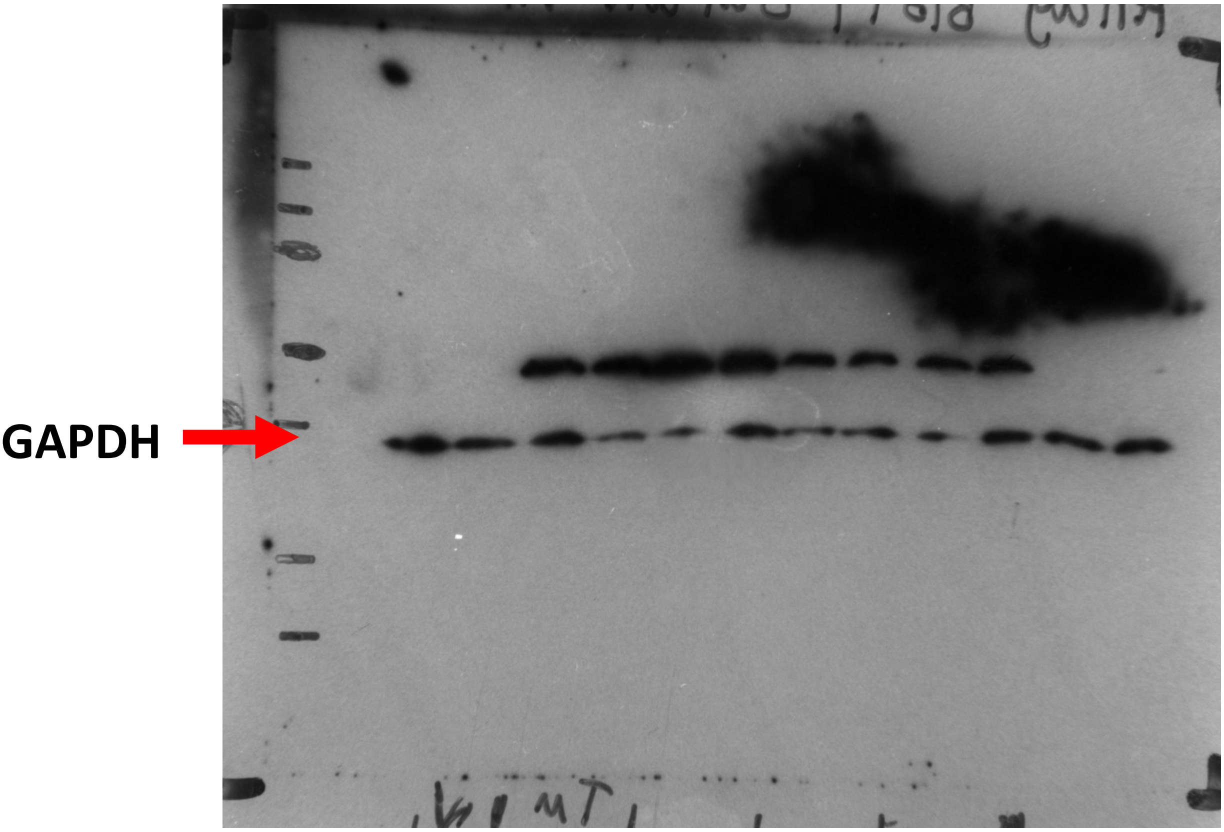

Western blots from Figure 3C

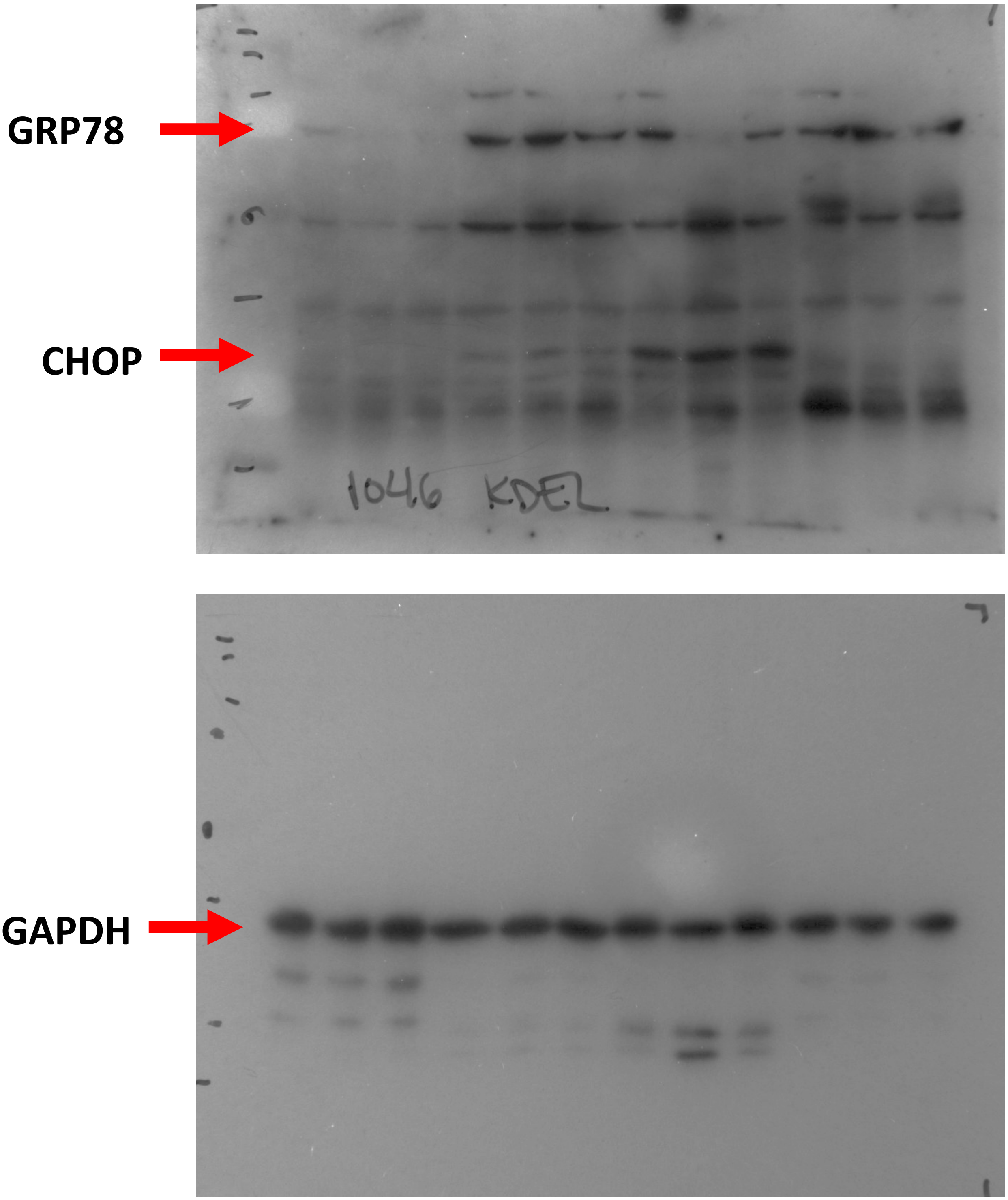

GRP78

CHOP

GAPDH

Western blots from Figure 5A

Acetyl H3 | **β-actin**

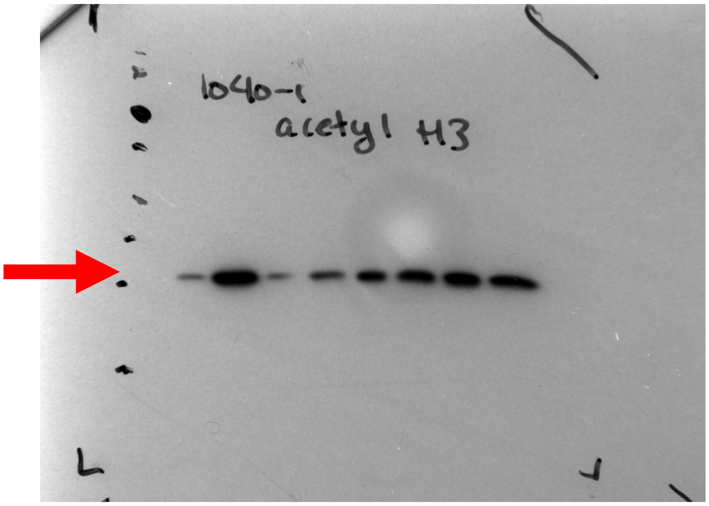

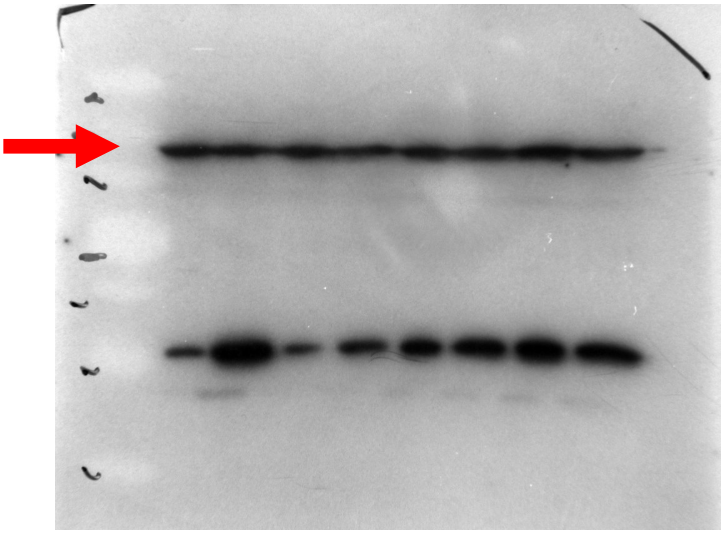

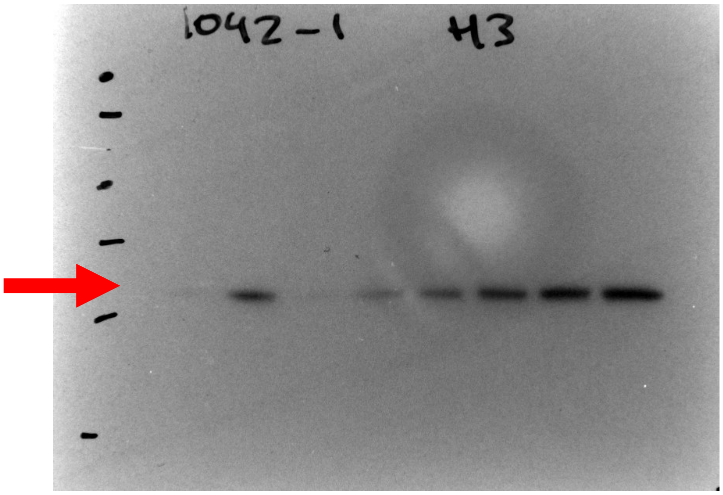

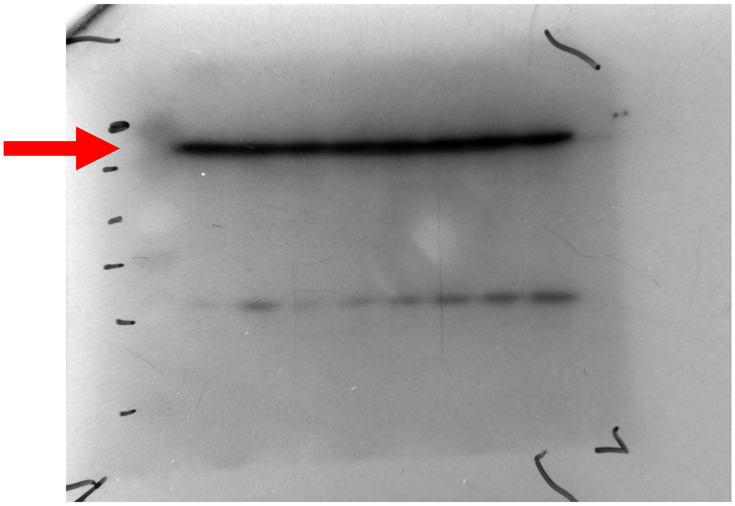

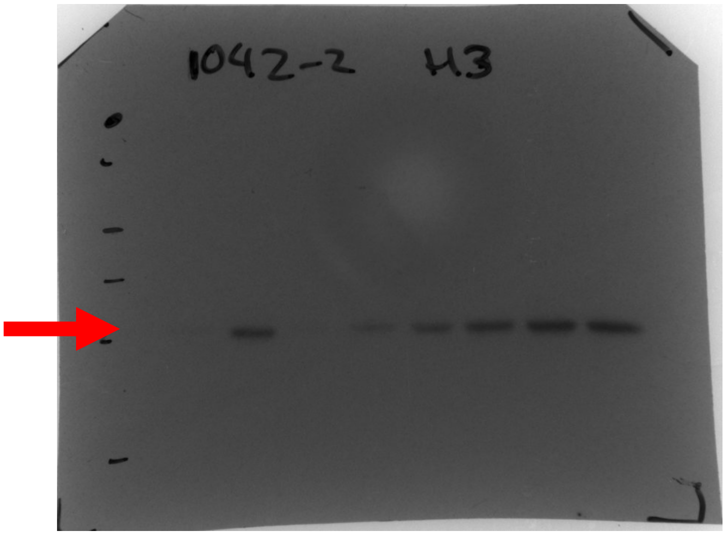

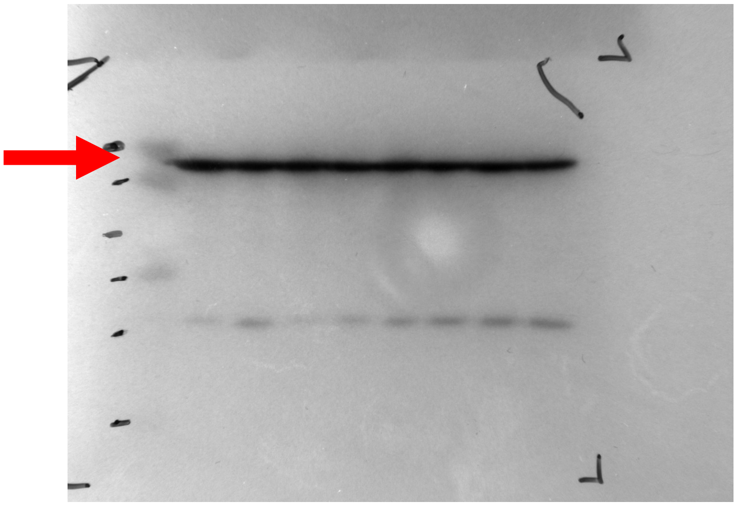

Western blots from Figure 5B

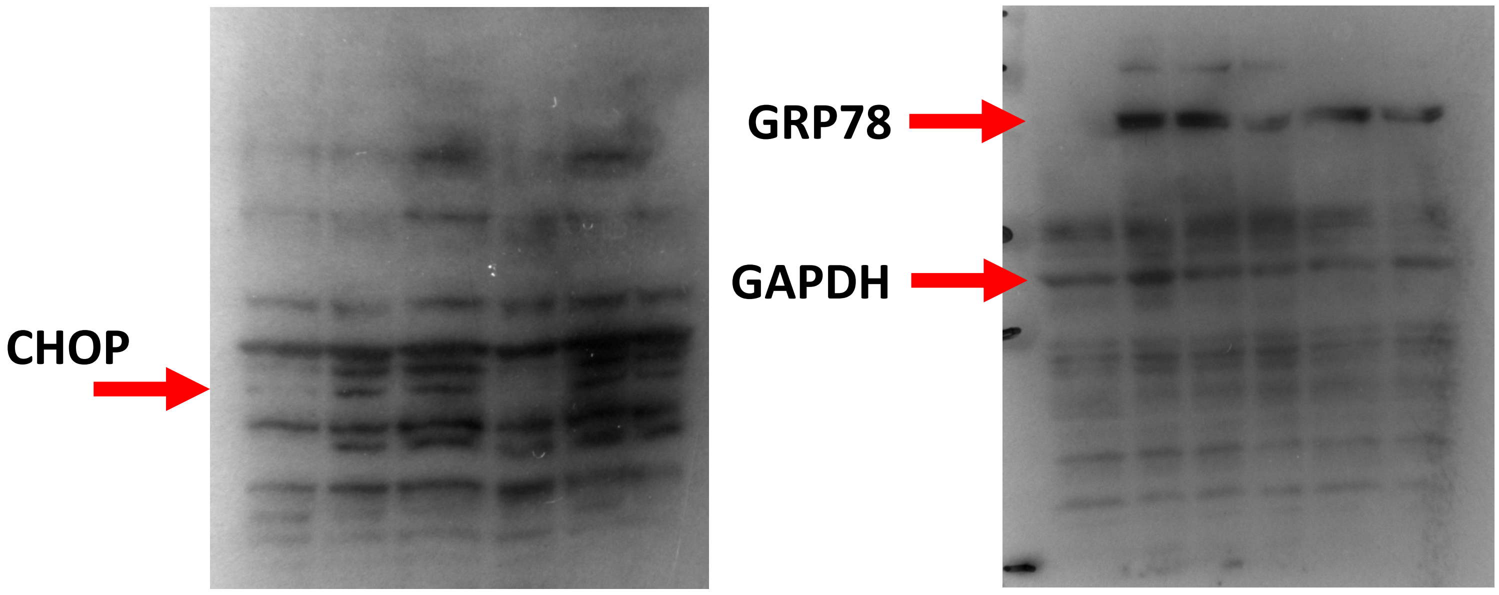

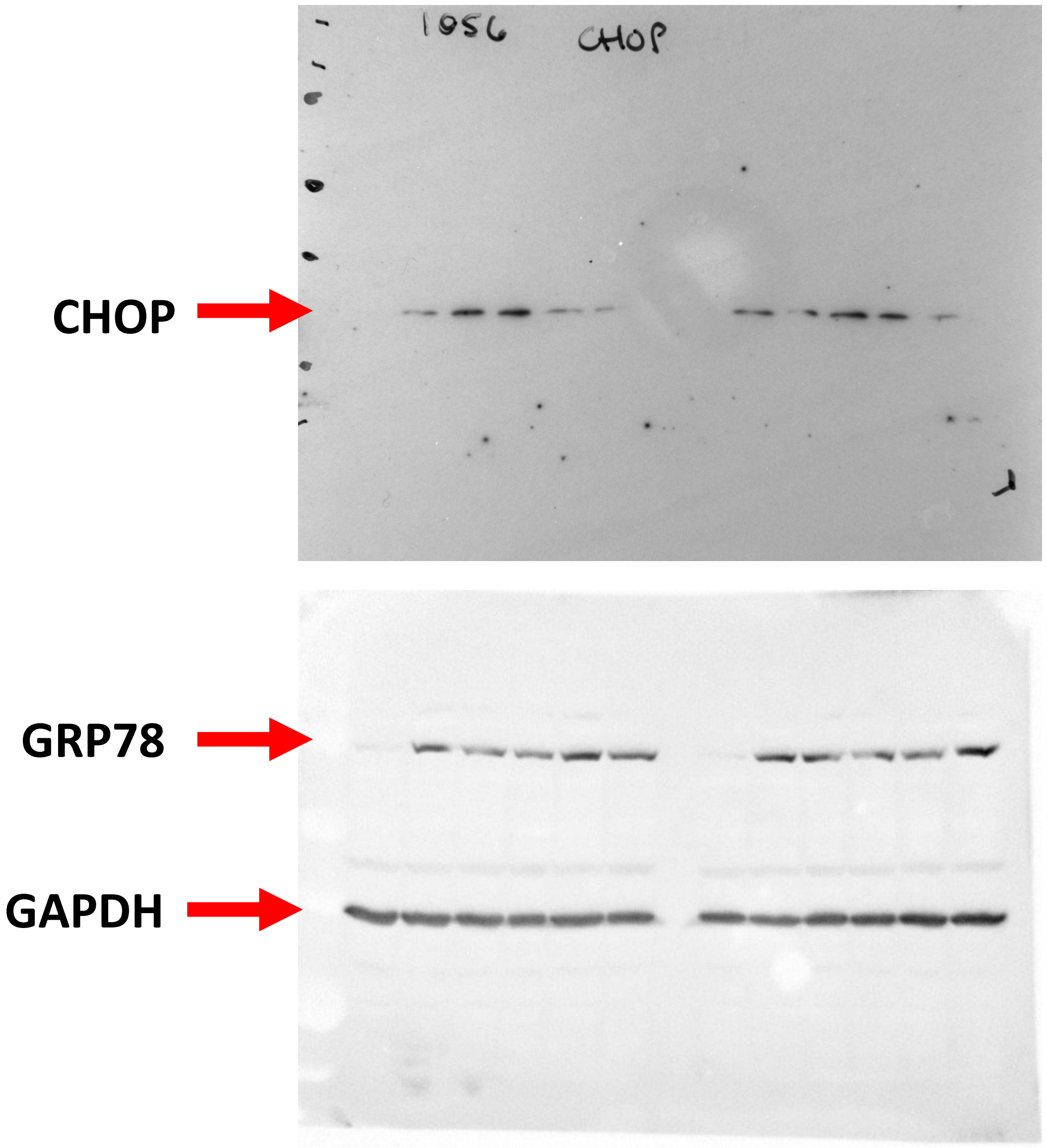

Supplement: S1 Raw images — (PDF) [file pone.0260519.s001.pdf]
